# Italian Dental Curriculum

**Published:** 1903-04-15

**Authors:** Vincenzo Guerini

**Affiliations:** Italy


### ITALIAN DENTAL CURRICULUM.

BY DR. VINCENZO GUERINI, ITALY.

Report to the International Commission of Education.

For about twelve years there has been a law in Italy which requires that all dentists must be holders of the degree of doctor of medicine before they can be allowed to practice their profession. This legislative measure presents great inconveniences, as I have on several occasions endeavored to demonstrate. I have pointed out the necessity that odontology should be taught as a separate and autonomous science, and not as a simple branch of general medicine. I found myself in the necessity of discussing this topic with the *medecins-dentistes*, and as soon as this was taken up I found that in order to defeat my adversaries the first thing I had to do was to recognize whatever truth could be found in their arguments, this being in my estimation the only way of clearly demonstrating the fallacy of their conclusions.

In the arguments advanced by the *medecins-dentistes* we find some truthful assertions, viz: That in order to practice dentistry upon a scientific basis it is necessary that the practitioner should possess a rather extensive medical education. As dentists are called upon to treat living organs, it is indispensable that they should thoroughly understand all the disturbances that may affect these organs, all the organic or extra-organic causes which may bring them about, and all the possible relations of dental and peridental diseases to the rest of the organism. If this be admitted, it follows as a rational consequence that the medical education of dentists must not be too limited nor too superficial, as in order to be in a position to understand thoroughly everthing concerning dental diseases and their relations to morbid conditions of the organism it is necessary to possess a large share of medical science. I am therefore convinced that dental schools will have to extend gradually the scope of medical teaching, as the extent of the medical branches taught in dental schools at present is certainly insufficient. As an example, I will quote from the report of Dr. Charles Godon, of Paris, in his work, "Evolution de l'Art Dentaire."

From this report we gather that in the Ecole Dentaire of Paris, about four-fifths of the total time is devoted to technical teaching and only on-fifth to the scientific and medical subjects, to the importance of which we are now referring. If we consider that the dental course covers three years—that is, about thirty-six months—it is easy to see that by this arrangement the entire medical education required from the dental student could be given him in seven months, including in this time vacations and holidays. Now, you know that medical students who devote from five to six years to the study of this science leave the school after this time with a very incomplete knowledge of medicine. The time devoted to the teaching of medical branches in dental schools is very limited, and therefore the student can acquire only a very limited and superficial knowledge, which will not help him to understand the pathology of the dental and peridental organs and the complex relations which exist between these parts and the rest of the organism from the points of view of the etiology, pathology and therapeutics of the disorders of which these organs may be the seat.

It is true that it has been decided to increase the course to four years, but, as far as I am aware, this fourth year will be devoted entirely to technical teaching; therefore even with a four years course the medical education of dentists will remain just as imperfect and insufficient as it is at preent. I believe in the necessity of extending the course to a five years one, and during this period the dental student should be taught both general medicine and dentistry. I believe, further, that the curriculum should be arranged in such a way that the student might be able to devote at least four hours a day to medical subjects and the remaining five or six to strictly technical subjects. Five or six hours a day during five years should be sufficient to make skillful dentists and on the other hand four hours a day devoted to medical subjects during five years should create dentists possessing a reasonable amount of medical education. They should have an amount of medical knowledge at least sufficient for the practice of their profession upon scientific basis.

Regarding the question whether medical subjects should be taught in the dental schools or in the medical schools, I will positively answer that the medical subjects to be taught to dentists should be given by teachers making a specialty of these subjects in their bearing on dentistry. The necessity that the medical education of dentists should be a special one is certainly evident. Let us take as an example, anatomy. On the bucco-facial region the anatomical training of the dentist should be more complete than that of the physician, just as, on the other hand, the anatomy of other regions could be suppressed from the curriculum or given in a more superficial way without at all injuring the dentist's education. For similar reasons, the teaching of other branches of the medical program in dental schools should differ from the teaching of the same branches in the medical school.

To summarize, we will say that for dental students the course should be altogether a special one, and its purpose should be to impart to the student without the slightest loss of time the greatest amount of medical knowledge that will be useful to him in the practice of his profession.

I also wish to call your attention to the question of *preliminary education.* Unfortunately, this question can not be treated in a general way, because of the great differences presented by the organization of primary or elementary education, especially of the secondary education, in different countries; notwithstanding this difference in organization, however, I believe that the total duration of the studies that dental students should take up before entering institutions of higher education should not vary a great deal in the different countries. This involves, say, about twelve years. I believe that the preliminary education of dental students should be the same as that required from the physician, the lawyer, the engineer, etc. With a preliminary education of this sort lasting twelve years it is possible to undertake the study of dentistry after having acquired a good literary and scientific education, comprising among other things the study of ancient and modern history, physics, chemistry, natural history, mathematics, languages, etc. All these studies are apt to develop the intelligence of the student and will serve the purpose of excellent preparation for the study of dentistry, permitting the dental student to assimilate easily the knowledge which will be imparted to him in the professional schools. The better educated the dentist is the more will he be respected. It is essential that he should cease to be inferior to the physician from lack of either general culture or scientific requirements. He should rise to the same level as the physician, and should be prepared to intelligently discuss with him any scientific subject. He should be able to write correctly and easily, in order to be in a position to increase the literature of the profession by contributing works on dental topics. It would be a shame for the profession, if the greatest part of its literature were written by physicians, and not by dentists, as has already been asserted,

For the foregoing reasons, I am led to express the wish that dental candidates should have a more complete preliminary education, and that in the curricula of dental schools more time should be devoted to the study of medical subjects.—*Cosmos.*

